# Homœopathy

**Published:** 1837-01

**Authors:** 


HOMŒOPATHY.

At a meeting of the French Academy of Medicine, held on the 27th of January, 1836, a letter was read from the minister of public instruction, requesting the opinion of the Academy on the propriety of the government permitting the establishment of a homœopathic hospital and dispensary. It will be necessary to inform our readers that at present it is illegal in France to form a society consisting of more than twenty persons, for any object whatever, without the sanction of the government. In consequence, the Homœopathic Society applied to the government to permit the immediate establishment of a dispensary, where all diseases should be treated gratuitously on the homœopathic system; and, secondly, that, when the Society had collected the necessary funds, a clinical hospital might be instituted, to teach the new system. The minister, thinking his decision involved not only a scientific opinion but an important question of medical police, referred the matter to the Academy, who appointed a committee to report upon it. MM. L'Herminier, Andral, and Lisfranc were some of those selected; and M. Adelon drew up the Report. As this is of some length, our space will only allow us to give a mere outline of it.

The committee state that, to form an absolute judgment, it would be necessary to read all the principal works on the subject, so as to submit the principles of the new system to a sound criticism, and to judge them *à priori,* guided by the experience in medicine of the last two thousand years; and also to verify the statements by experiment. The first might be accomplished, but the second could not possibly in the time allotted to the committee. Fortunately, however, it is not necessary to undertake this difficult task in order to answer the question made by the minister, whose demand refers to homœopathy relatively to medical police. As hospitals are for the gratuitous relief of the poor, and as these institutions are under the immediate superintendence of the government, it is necessary that it should be assured of their utility. It is therefore as if the minister asked, "Is homœopathy so universally approved that I may share in the responsibility of its exclusive application in the way proposed? Can I present it to the poor of Paris with the certainty it will be always useful, and never hurtful?" If put in this form, the question can be answered without difficulty. The committee are of opinion that the proofs of the doctrine of homœopathy are far from producing conviction. Looked at in every point of view, it is doubtful. Judging it theoretically, many of its dogmas are contradictory, contrary to sound logic, and opposed to the experience of the majority of physicians for ages; the principles are at least still matters for controversy. Considered practically, the proofs are still to be made, and, if advisable, would require time, and the concurrence of the profession generally. In a word, the homœopathic doctrines, instead of being demonstrated, are yet to be studied; and consequently the government cannot be advised to take upon themselves the responsibility of its application.

On receiving this Report, the Academy decided that it was too reserved and too temperate in its opinion of homœopathy, and determined on an answer to the minister repelling it as a dangerous mode of treatment, and the offspring of quackery.

*Annales d'Hygiène publique et de Médecine Légale. Avril,* 1836.

---

